# An Approach for the Visualization of Temperature Distribution in Tissues According to Changes in Ultrasonic Backscattered Energy

**DOI:** 10.1155/2013/682827

**Published:** 2013-10-24

**Authors:** Jingjing Xia, Qiang Li, Hao-Li Liu, Wen-Shiang Chen, Po-Hsiang Tsui

**Affiliations:** ^1^School of Electronic Information Engineering, Tianjin University, Tianjin 300072, China; ^2^Department of Electrical Engineering, Chang-Gung University, Taoyuan 33302, Taiwan; ^3^Department of Physical Medicine and Rehabilitation, National Taiwan University Hospital and College of Medicine, Taipei 10002, Taiwan; ^4^Department of Medical Imaging and Radiological Sciences, College of Medicine, Chang-Gung University, Taoyuan 33302, Taiwan; ^5^Healthy Aging Research Center, Chang-Gung University, Taoyuan 33302, Taiwan

## Abstract

Previous studies developed ultrasound temperature-imaging methods based on changes in backscattered energy (CBE) to monitor variations in temperature during hyperthermia. In conventional CBE imaging, tracking and compensation of the echo shift due to temperature increase need to be done. Moreover, the CBE image does not enable visualization of the temperature distribution in tissues during nonuniform heating, which limits its clinical application in guidance of tissue ablation treatment. In this study, we investigated a CBE imaging method based on the sliding window technique and the polynomial approximation of the integrated CBE (ICBE_pa_ image) to overcome the difficulties of conventional CBE imaging. We conducted experiments with tissue samples of pork tenderloin ablated by microwave irradiation to validate the feasibility of the proposed method. During ablation, the raw backscattered signals were acquired using an ultrasound scanner for B-mode and ICBE_pa_ imaging. The experimental results showed that the proposed ICBE_pa_ image can visualize the temperature distribution in a tissue with a very good contrast. Moreover, tracking and compensation of the echo shift were not necessary when using the ICBE_pa_ image to visualize the temperature profile. The experimental findings suggested that the ICBE_pa_ image, a new CBE imaging method, has a great potential in CBE-based imaging of hyperthermia and other thermal therapies.

## 1. Introduction

Previous studies have shown that hyperthermia complements chemotherapy and radiotherapy, increasing the success of cancer treatment [1–3]. When using hyperthermia, monitoring temperature is essential to ensure accurate and appropriate thermal dosage. The development of temperature-imaging techniques to measure the distribution of temperature has, therefore, been a long-term critical research goal.

Magnetic resonance imaging (MRI) is currently the standard imaging method used to monitor temperature changes in tissues [4, 5]. Previous studies have shown that MRI can provide satisfactory spatial resolution with a temperature accuracy of 1°C. However, imaging temperature variations in heated regions using MRI might be difficult in practice because of the requirements for significant capital investment and the development of compatible heating therapies [6]. Compared to MRI, ultrasound imaging provides a convenient and powerful tool because of low cost, use of nonionizing radiation, simple signal processing, and real-time capability. Ultrasound imaging might, therefore, provide a more appropriate option for the clinical monitoring of temperature distributions. 

The frequently used acoustic parameters for the monitoring of temperature include (1) echo shifts caused by changes in tissue thermal expansion and speed of sound [7, 8], (2) variations in acoustic attenuation [9], and (3) changes in the backscattered energy (CBE) of ultrasound [10, 11]. Each method has specific temperature sensitivities, applications, and limitations. Previous studies identified that the CBE, in comparison with echo shifts and attenuation, is nearly monotonic in the clinical hyperthermia temperature range [10–12]. Considering the clinical value of imaging tools that visually display temperature profiles in tissues, investigators further proposed the CBE image based on a parametric mapping of CBEs as an ultrasound temperature-imaging method for the monitoring of regions undergoing heating [6, 13, 14]. The recent literature has confirmed the usefulness of the CBE image for detecting variations in tissue temperatures.

In practice, use of the CBE image for temperature estimation may have some difficulties. First, the CBE image is a ratio map obtained from the envelope image divided by the reference envelope image on a pixel-to-pixel basis [13]. Because temperature change induces the displacement of image features [13], the tracking of pixels between images obtained at adjacent temperatures is necessary to obtain the correct CBE at each pixel. Motion tracking and compensation are typically the most computationally intensive components of temperature imaging and hinder its real-time implementation [7, 8, 15, 16]. On the other hand, increasing temperature might also increase the degree of acoustic nonlinearity [17]. In this condition, local waveforms of the received radio-frequency (RF) signals can differ, resulting in greater error when performing pixel-by-pixel division between two images following motion tracking and compensation [18]. In addition, the CBE image cannot clearly illustrate the contours of temperature distribution in a tissue during nonuniform heating by high-intensity focused ultrasound (HIFU) or microwave ablation. This is because the CBE image behaves in the same manner as a speckle image, reducing sensitivity and contrast for illustration of the temperature profile.

Clinical application of CBE imaging during hyperthermia and other thermal therapies might, therefore, require a different imaging method for guidance of the tissue ablation process based on the CBE concept. This study investigated a new CBE imaging method based on the mathematic polynomial approximation of the integrated CBE (ICBE_pa_) estimated using the sliding window technique, which have resolved the limitations of the conventional CBE image. 

In the next section, we introduce the theoretical background of the CBE image and present the concept and details of the new CBE imaging method. Then, we describe the experimental procedures used to validate the feasibility of the proposed method. The results are presented for discussion. The impact and contributions of this study are summarized in conclusion. 

## 2. Methods

### 2.1. Conventional CBE Imaging Method

Previous studies have extensively investigated the temperature dependence of the CBE and the CBE imaging algorithm [6, 10–14]. According to the above literatures, we briefly review the principle of the CBE. Changes in backscattered energy with temperature are primarily caused by thermal effects on the backscatter coefficient. The temperature dependence of the backscattered energy can be simplified by normalizing it to the baseline value obtained at a reference temperature (typically 37°C), removing the influence of factors with little or no temperature dependence. The CBE, as a function of temperature for a single scatterer, can then be approximated as the ratio of the temperature-dependent backscatter coefficients (*η* values) at temperature *T* and reference temperature *T*_*R*_:

(1) η(T)η(TR)=((ρmc(T)m2−ρsc(T)s2ρsc(T)s2)2+13(3ρs−3ρm2ρs+ρm)2)×((ρmc(TR)m2−ρsc(TR)s2ρsc(TR)s2)2+13(3ρs−3ρm2ρs+ρm)2)−1,

where *ρ* is the mass density, *c*(*T*) is the temperature-dependent sound speed, and the *m* and *s* subscripts refer to the medium and scatterer, respectively. From the model described in (1), it can be predicted that the backscattered energy contributed by lipid-based scatterers would increase with increasing temperature, whereas that corresponding to aqueous scatterers would decrease [10–12].

The algorithmic procedure used to form the CBE temperature image has been described previously [13, 14]. In brief, the speckle motions (echo shift) caused by changes in sound speed and tissue thermal expansion were tracked and compensated by maximizing the cross-correlation between images obtained at adjacent temperatures. Optimization and image resampling were performed to eliminate the dependence of the image on the spatial sampling period. Envelope data of the compensated images at each temperature were obtained using Hilbert transform. Envelope values were squared to determine the backscattered energy. The CBE image was then obtained by calculating the ratio of the backscattered energy at each temperature relative to the reference at each pixel.

### 2.2. Proposed CBE Imaging Method

The proposed algorithm for CBE imaging first used a square window within the envelope image at temperature *T* to collect the regional backscattered envelopes *h*(*T*). If *E*[·] denotes the statistical mean, then the regional CBE value (in decibels; dB), calculated using the data acquired by the window (CBE_*w*_), compared to the reference temperature *T*_*R*_, can be calculated using

(2) $$\begin{array}{c} {\text{CBE}}_{w}=10\cdot \text{lo}{\text{g}}_{10}\left(\frac{E\left[h{\left(T\right)}^{2}\right]}{E\left[h{\left(T_{R}\right)}^{2}\right]}\right). \end{array}$$

The regional CBE_*w*_ was assigned as the new pixel located in the center of the window. The described process was repeated with the window moving throughout the entire envelope image in steps of a certain number of pixels determined by the window overlap ratio (WOR), yielding the CBE_*w*_ image as the map of regional CBE_*w*_ values. 

The absolute value map of the CBE_*w*_ image was then used as the integrated CBE (ICBE) image. The ICBE map needs further processing for visualization of temperature distributions and heat transfer in ablated tissue during nonuniform heating. Previous studies have extensively used the mathematic polynomial approximation to fit experimental data and predict temperature distribution and heat conduction behavior [19–21]. This study applied the polynomial approximation during ICBE image processing. Suppose that the original ICBE image prior to smoothing is *X*_*i*,*j*_, where *i* and *j* are indices of the image depth and width, respectively. We assumed that the function in each direction was a polynomial of order *p*. Because *p* is much smaller than the number of pixels in the axial and lateral directions, the *X*_*i*,*j*_ data were used to determine the optimal polynomial using the least-squares method. *X*_*i*,*j*_ was replaced with the value calculated by the optimal polynomial. This approximation was performed along each line in the axial and lateral direction, with *f*_*p*_(·; *V*) being the optimal polynomial of order *p* reconstructed from the vector *V* = (*V*_1_, *V*_2_, …, *V*_*n*_) located at indices 1,2,…, *n*. After applying polynomial approximations in the lateral and axial directions, image ${\overset{-}{X}}_{i,j}^{}$ was constructed using the following two procedures:

(3) $$\begin{array}{c} X_{i,j}^{*}=f_{p}\left(i;X_{1:n_{d},j}\right),\;\text{for}\;\;\text{each}\;\;j,\\ {\overset{-}{X}}_{i,j}^{}=f_{p}\left(j;X_{i,1:n_{w}}^{*}\right),\;\text{for}\;\;\text{each}\;\;i, \end{array}$$

where *n*_*d*_ and *n*_*w*_ are the numbers of pixels in the axial (depth) and lateral (width) directions, respectively, *X*_1:*n*_*d*_,*j*_ is the vector *X*_1,*j*_, *X*_2,*j*_,…, *X*_*n*_*d*_,*j*_,  *X*_*i*,j_* is the intermediate image, and ${\overset{-}{X}}_{i,j}^{}$ is the ICBE_pa_ image.

## 3. Experimental Verification

### 3.1. Ablation Experiments

**Figure 1:**
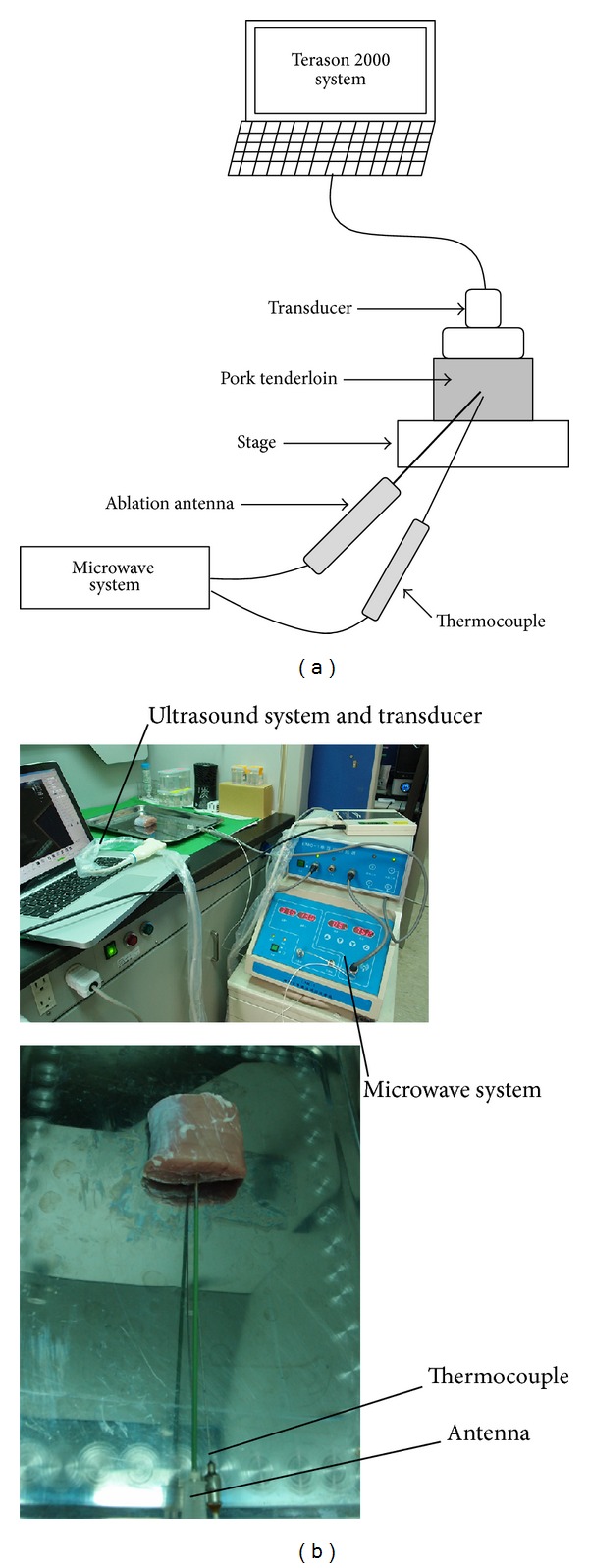
(a) Schematic diagram of the experimental setup. (b) A real representation of the experimental setup.

The feasibility of the proposed method to monitor the distribution of temperature during nonuniform heating was evaluated by conducting tissue ablation experiments on excised tissues. The tissue sample was prepared from pork tenderloin extracted from the psoas major muscle along the central spine. Tissue ablation was conducted using a microwave delivery system (UMC-1, Chinese PLA General Hospital, Institute 207 of the Aerospace Industry Company, Beijing, China) that operates at a frequency of 2.45 GHz and has electrical power ranging from 1 to 99 W. The tenderloin sample was preheated by a temperature-regulated water tank to an initial reference temperature of 37°C. The microwave antenna was then inserted into the sample for irradiation. Ablation treatment performed at 2 W for 420 s produced significant and stable increases in temperature. A commercial ultrasound scanner (Model 2000, Terason, Burlington, MA, USA) that can output raw RF signals digitized at a sampling rate of 30 MHz was used to image the tissue during heating. A wideband linear array probe (Model 10L5, Terason) with a central frequency of approximately 7 MHz was used. A pulse echo test of the transducer showed that the pulse length was 0.7 mm. The image raw data were acquired every 20 s. Each image consisted of 128 scan lines of backscattered signals, and Hilbert transform was applied to each scan line to obtain the corresponding envelope image. The temperature as a function of ablation time was measured using a thermocouple that was attached to the microwave antenna. Measurements of five tissue samples were performed. The experimental setup is shown in Figure 1.

### 3.2. Data Analysis

The envelope signals obtained from tissue samples were used for B-mode and CBE imaging. The B-mode image formation was based on the log-compressed envelopes with a dynamic range of 60 dB. A sliding square window with side lengths corresponding to one pulse length (0.7 mm) was selected to construct the CBE_*w*_, ICBE, and ICBE_pa_ images. To evaluate the performances of the new CBE image in temperature estimation, the image data were used to calculate the pixel magnitude as a function of heating time. Moreover, the contrast-to-noise ratio (CNR) was calculated as an estimate of the contrast resolution, defined as

(4) $$\begin{array}{c} \text{CNR}=\left|\frac{\mu_{\text{ablation}\;\text{region}}-\mu_{\text{background}}}{\sigma_{\text{ablation}\;\text{region}}+\sigma_{\text{background}}}\right|, \end{array}$$

where *μ* and *σ* are the mean and standard deviation of the pixel values in the images, respectively. The above quantitative data analyses were based on data acquired from the regions of interest (ROI) located in the background and the ablation zone. The sizes of the ROI were 5 mm × 5 mm.

## 4. Results

**Figure 2:**
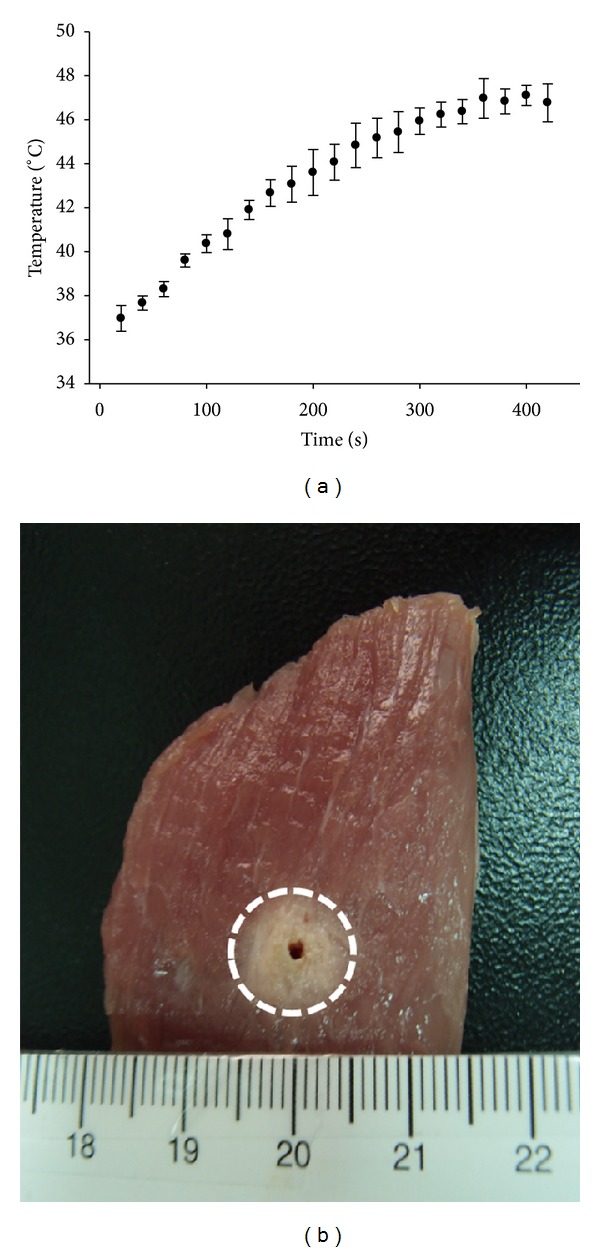
Temperature measurement during microwave ablation and the typical image of tissue section after ablation.

**Figure 3:**
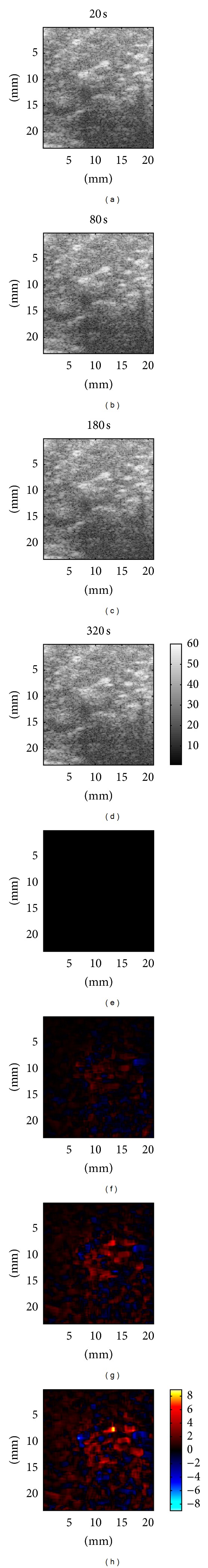
((a)–(d)) Typical B-mode images of pork tenderloin obtained at different heating times and the corresponding ((e)–(h)) CBE_*w*_ images.

**Figure 4:**
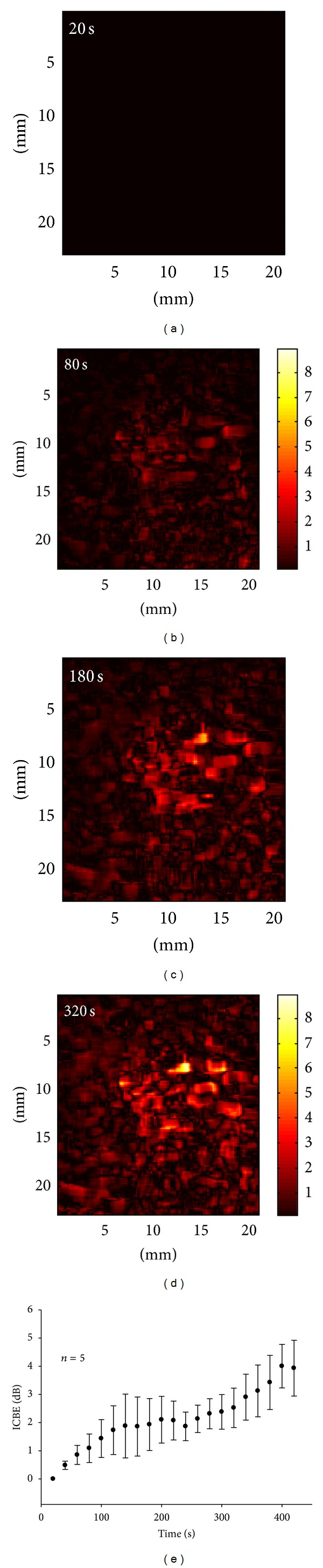
((a)–(d)) Typical ICBE images obtained at different heating times; (e) ICBE values as a function of heating time.

Figure 2 shows the temperature as a function of ablation time and the typical image for the cross-section of pork tenderloin after microwave ablation. The temperature in the ablation region surrounding the antenna increased from approximately 37°C to 46°C during heating. In the end of ablation, a tissue denaturation region with a roughly circular shape with a diameter of about 1 cm was formed in the tissue background.  Figure 3 shows typical B-scans of pork tenderloin obtained during microwave ablation and the corresponding CBE_*w*_ images (a WOR of 80% was used). The brightness of the red-blue interlaced shading in the CBE_*w*_ image gradually increased with increasing temperature.  Figure 4 shows the results of the ICBE image and the average ICBE as a function of heating time. The brightness of the ICBE image increased during heating, corresponding to the average ICBE value increase from 0 dB to 4 dB.

**Figure 5:**
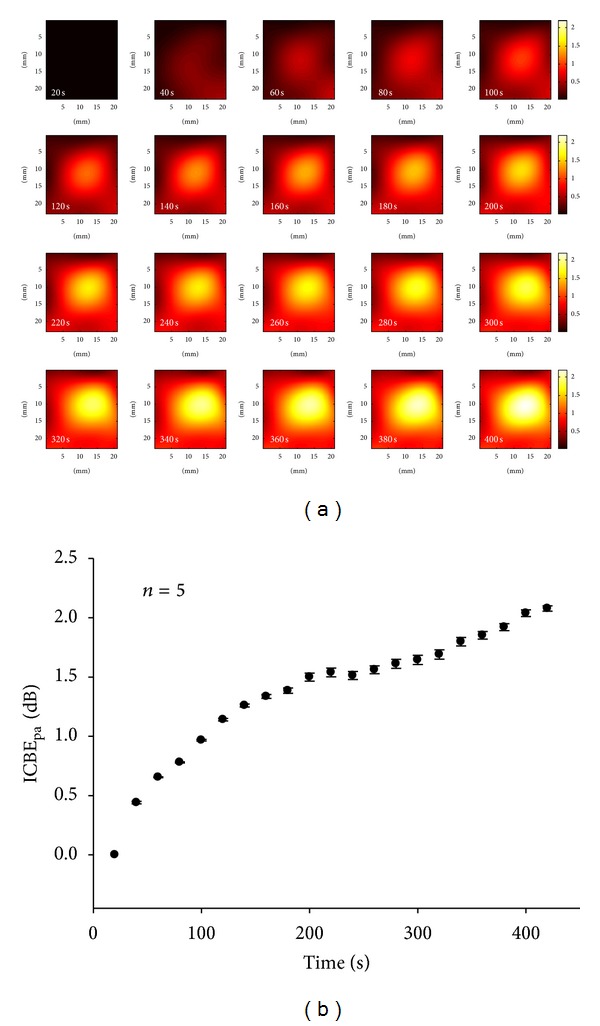
(a) Examples of ICBE_pa_ images of pork tenderloin obtained at different heating times; (b) ICBE_pa_ values obtained in the tissue ablation experiments as a function of heating time.

**Figure 6:**
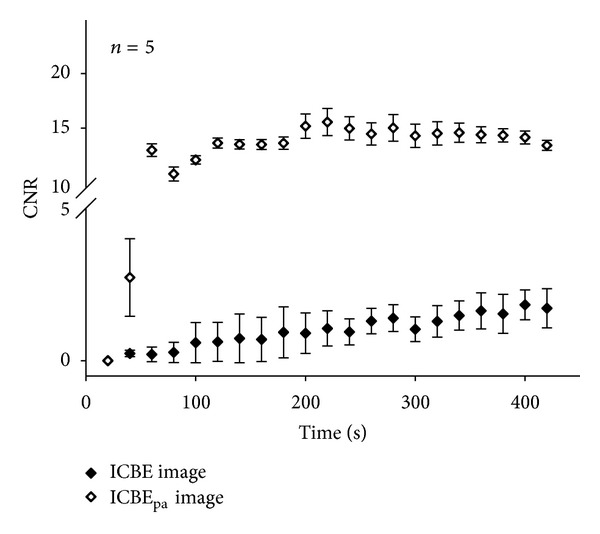
Contrast-to-noise ratios as functions of heating times for ICBE and ICBE_pa_ images.

Figure 5(a) shows the results of the ICBE_pa_ images (using order 7). We observed that the brightness of the ICBE_pa_ image gradually increased during heating for 420 s, with the average ICBE_pa_ value increasing from 0 dB to approximately 2 dB, as shown in Figure 5(b). This demonstrated that the ICBE_pa_ image can visualize the temperature distribution in the tissue. To evaluate the enhancement of the contrast after applying polynomial approximation, the curves of the CNR were plotted as a function of heating time in Figure 6. The dynamic range of the CNR during the heating for the ICBE image was approximately 1.6. In contrast, the dynamic range of the CNR for the ICBE_pa_ image was 13.4. The ICBE_pa_ image, thus, allows the temperature profile to be visualized with excellent image contrast.

**Figure 7:**
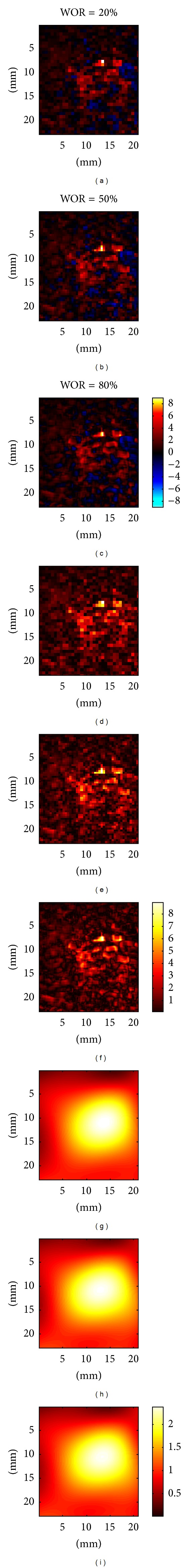
Examples of ((a)–(c)) CBE_*w*_, ((d)–(f)) ICBE, and ((g)–(i)) ICBE_pa_ images of pork tenderloin postheating for 400 s, constructed using WORs of 20%, 50%, and 80%, respectively.

**Figure 8:**
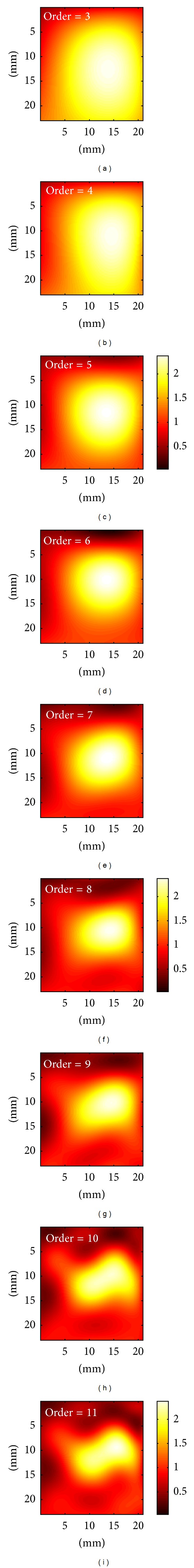
((a)–(i)) Examples of ICBE_pa_ images of pork tenderloin postheating for 400 s, constructed using polynomial approximations of different orders.

Figure 7 shows examples of the CBE_*w*_, ICBE, and ICBE_pa_ images postheating for 400 s, using WORs of 20%, 50%, and 80%, respectively. We observed that a lower WOR degraded the resolutions of the CBE_*w*_ and ICBE images because of the use of fewer pixels to form the image. However, the features and patterns of the ICBE_pa_ image did not exhibit significant changes, with the ICBE_pa_ image still well-describing the contour of the temperature distribution.  Figure 8 displays ICBE_pa_ images of pork tenderloin obtained postheating for 400 s, using polynomial approximations of different orders. Compared with the cross-sections of pork tenderloin shown in Figure 2, the ICBE_pa_ image might overestimate the temperature distribution when using lower order polynomial approximations. The ICBE_pa_ image might reflect the actual temperature distribution when using polynomial approximations of orders of 6 to 7, whereas using higher order polynomial approximations might underestimate the range of the temperature distribution.

## 5. Discussion

### 5.1. The Significance of This Study

Implementing real-time temperature imaging based on the CBE concept in different clinical applications requires the development of a new CBE imaging method to overcome the limitations of conventional CBE imaging. In our opinion, CBE imaging for clinical purposes should have two essential features: (i) reduced dependency of the image performance on echo shift tracking and compensation, with no requirement for echo shift compensation being the objective, and (ii) the ability to visualize the contour of temperature distribution during nonuniform heating, enabling its application in guidance of tissue ablation. The current results have demonstrated that the proposed CBE imaging method fulfills the above two requirements. 

### 5.2. Window Size for Constructing the Proposed CBE Imaging

In our proposed CBE imaging method, the algorithm replaces the pixel-to-pixel calculation with a “region-to-region” calculation. The reason is that using data within a windowed region reflected average trend of backscattered energy, which may reduce the influence of echo shifts and waveform distortion due to heating tissues. Under this assumption, how to select an appropriate window size for regional CBE computation is a key determination to implement visualization of temperature distributions in the absence of echo shift tracking and compensation. According to the analysis by Seip and Ebbini [22], the movement of a scatterer caused by thermal expansion when the temperature increases from 37°C to 50°C is typically less than 2 *μ*m. Over this temperature range, the maximum speckle motion caused by changes in the sound speed for different types of tissues is approximately 0.5 mm in the axial and lateral directions [13]. In this study, the side length of the square window used for regional CBE computation was determined by the pulse length of the used transducer, which is larger than the maximum echo shift between 37°C and 50°C revealed in the previous study. In this circumstance, the resolution of the CBE image constructed using the sliding window technique is actually not enough to describe the behavior of echo shift. This may be the reason why our CBE imaging methodology can work without echo shift tracking and compensation.

### 5.3. Polynomial Approximation of the CBE Image

The idea for the proposed algorithmic procedure is similar to the concept of ultrasound Doppler imaging. We can better understand the spirit of the new CBE imaging method from the comparison with Doppler ultrasound. The Doppler shift signals in color Doppler ultrasound imaging are presented in color and superimposed on grayscale images to reflect blood flow information associated with velocity and direction. Limitations of the color Doppler ultrasound image include angle dependence, aliasing, and insensitivity to slow flows [23]. These limitations are less significant in power Doppler images that include positive and negative components of the Doppler shift signals [23, 24].

As discussed, the ultrasound CBE increases with increasing temperature for lipid-based scatterers and decreases with increasing temperature for aqueous scatterers. Similar to the shadings of color Doppler ultrasound corresponding to different flow directions, the conventional CBE image typically also has two shadings: one for lipid-based scatterers (increasing CBE) and the other for aqueous scatterers (decreasing CBE). However, these two shading types can be ambiguous if CBE imaging is performed without echo shift tracking and compensation. This shading ambiguity could be treated as the aliasing effect of the CBE image, which is similar to the aliasing effect caused by an insufficient pulse repetition rate in color Doppler ultrasound. Like the concept of power Doppler ultrasound, the ICBE image is formed by integrating the positive and negative CBE values as the strengths. In this condition, the ICBE image cannot reflect the properties of scatterers in a tissue (lipid-based or aqueous). Nevertheless, it may be treated as an aliasing-independent CBE image to provide a better sensitivity and contrast for the depiction of temperature distribution in a nonuniform heating region.

However, we found that the speckle-like features in the ICBE image still hinder improving the performance of temperature profile visualization. The ICBE image has the ability to describe the temperature distribution in ablated tissues, but it did not have a relatively high CNR. In the contrary, the speckle-free ICBE_pa_ image had a larger CNR, demonstrating that the polynomial approximation provides the ICBE image with the ability to highlight the region and location of ablation by reflecting temperature information with very good contrast. 

### 5.4. Considerations on Performing Polynomial Approximation of the CBE Image

Some aspects of the algorithmic parameters for construction of the ICBE_pa_ image require further discussion to point out some considerations in practice. At first, our results demonstrated that the WOR did not significantly affect the ICBE_pa_ image. This suggested that constructing the ICBE_pa_ temperature image using a lower WOR is feasible to reduce the computational load. Second, the order selection of conducting polynomial approximation would affect the accuracy of estimations of the effective range and size of the temperature profile for the transfer of heat in ablated tissue [25]. This difficulty can be confirmed by our experimental results, indicating that using an inappropriate order for the polynomial approximation can result in overestimation or underestimation of the temperature profile. Some previous studies have also shown that using a polynomial might not be robust to outliers, which can cause fitting errors in ultrasound data [26, 27]. Future studies should aim to explore the optimal polynomial approximation for producing clinical ICBE_pa_ images.

### 5.5. Limitations and Future Work

For temperature elevations higher than 45°C, the major limitation of ultrasound temperature estimation comes from the irreversible changes in the acoustic properties of tissue caused by necrosis. This may explain why the image parameter and the temperature do not have a linear relationship. On the other hand, the proposed CBE image is dedicated to the visualization of the temperature distribution in a tissue. Absolute temperature values are unavailable from the reading of the proposed CBE image. However, this limitation may be overcome by establishing a calibration table for further applications of temperature measurements.

It is worth noting that the construction of the proposed CBE imaging method just needs raw RF signals acquired from a standard pulse-echo ultrasound system and does not require echo shift compensation. This implies that the algorithm of the proposed CBE imaging method can be combined with most commercial ultrasound systems, making it possible to implement real-time temperature imaging. However, the frame rate of real-time temperature imaging is difficult to estimate in the current stage, because the computational efficiency may depend on system specifications and programming skills. In future developments, we would suggest using hardware and parallel processing techniques as the algorithmic kernel to make the frame rate of temperature imaging close to that of ultrasound B-scan. 

## 6. Conclusion

In this study, we have proposed a new CBE imaging method based on the combination of the sliding window technique and the polynomial approximation (i.e., ICBE_pa_ image) to successfully implement the visualization of temperature distribution in the ablated tissue. The ICBE_pa_ approach is an aliasing-independent and speckle-free temperature image that visualizes temperature profile with no requirement for echo shift tracking and compensation, indicating the potential clinical application of CBE imaging in guidance of tissue ablation and other thermal therapies.
